# The Influence of Medical Professional Knowledge on Empathy for Pain: Evidence From fNIRS

**DOI:** 10.3389/fpsyg.2018.01089

**Published:** 2018-07-17

**Authors:** Jingdan Xie, Haibo Yang, Xiaokai Xia, Shengyuan Yu

**Affiliations:** ^1^International Headache Center, Department of Neurology, Chinese PLA General Hospital, Beijing, China; ^2^Academy of Psychology and Behavior, Tianjin Normal University, Tianjin, China

**Keywords:** medical professional knowledge, empathy, pain, fNIRS, dorsolateral prefrontal cortex (DLPFC)

## Abstract

Empathy is a mental ability that allows one person to understand the mental and emotional state of another and determines how to effectively respond to that person. When a person receives cues that another person is in pain, neural pain circuits within the brain are activated. Studies have shown that compared with non-medical staff, medical practitioners present lower empathy for pain in medical scenarios, but the mechanism of this phenomenon remains in dispute. This work investigates whether the neural correlates of empathic processes of pain are altered by professional medical knowledge. The participants were 16 medical students who were enrolled at a Chinese medical college and 16 non-medical students who were enrolled at a normal university. Participants were scanned by functional near-infrared spectroscopy while watching pictures of medical scenarios that were either painful or neutral situations. Subjects were asked to evaluate the pain intensity supposedly felt by the model in the stimulus displays, and the Interpersonal Reactivity Index-C (IRI-C) questionnaire was used to measure the empathic ability of participants. The results showed that there is no significant difference between medical professional and non-medical professional subjects in IRI-C questionnaire scores. The subjects of medical professions rated the pain degree of medical pictures significantly lower than those of non-medical professions. The activation areas in non-medical subjects were mainly located in the dorsolateral prefrontal cortex, frontal polar regions, posterior part of the inferior frontal gyrus, supramarginal gyrus, supplementary somatosensory cortex and angular gyrus, whereas there was a wide range of activation in the prefrontal lobe region in addition to the somatosensory cortex in medical professionals. These results indicate that the process of pain empathy in medical settings is influenced by medical professional knowledge.

## Introduction

Our perception of others not only involves understanding the emotional experiences of other individuals but also generates a similar emotional state. The ability to feel and share the emotional experiences of another is known as empathy (Ungerer et al., 1990; Preston and de Waal, 2002). Empathy is the mental ability that allows one person to infer the mental and emotional states of others and determines how to effectively respond to that person. This ability plays an important role in successful interactions in a social context (Gu and Han, 2007; van Heck et al., 2017). When a person receives cues that another person is in pain, neural pain circuits are activated, as if experiencing one’s own pain (Danziger et al., 2006; Bernhardt and Singer, 2012; Meng et al., 2012). An individual who sees or imagines others suffering from pain will feel uncomfortable with sympathy and concern, among other things. As a complex social psychological phenomenon, empathy for pain can help people avoid risks and danger, establish good interpersonal relationships, and promote pro-social behaviors, which are of great significance to human existence and reproduction (Wu et al., 2017).

Several neuropsychological studies have indicated that empathy is engaged in the cognitive and affective processes. Watching disgust expression or pain expression increased activity in the anterior insula and the anterior cingulate cortex (ACC) (Saarela et al., 2007). Other studies showed that observing others being pricked reduced amplitudes of motor-evoked potentials, suggesting that the sensorimotor cortex is related to empathic processing (Avenanti et al., 2005, 2006). These neuroimaging findings suggest that the experience of one’s own emotion and empathic responses to the emotions of others may share common neural mechanisms (Gu and Han, 2007).

Empathy also plays an important role in medical settings. It is generally believed that when medical workers can understand the pain of patients, this understanding is conducive to establishing a harmonious doctor–patient relationship (Smith et al., 2017). Studies have indicated that doctors in the clinical environment show less empathy (Roter et al., 1997). In particular, empathy was found to be significantly lower among medical professionals than among non-medical ones (Decety and Lamm, 2006; Smith et al., 2017). Studies involving medical students have revealed that over the years of medical education or work experience, levels of empathy in medical students change (Roter et al., 1997; Ahrweiler et al., 2014). For example, one study in which therapists and non-medical staff were invited to assess the degree of pain in acupuncture pictures, the rating given by therapists was significantly lower than that given by non-medical staff (Cheng et al., 2007). However, other studies have provided an opposite conclusion. Kataoka et al. (2009) demonstrated that empathy in medical students in Japan gradually increased with their enrollment time.

Some studies suggest that medical professional knowledge and personal experience are responsible for the differences in empathy between medical and non-medical staff (Roter et al., 1997; Suchman et al., 1997; Winseman et al., 2009; Ahrweiler et al., 2014). Gleichgerrcht and Decety (2013) used the Interpersonal Reactivity Index (IRI) questionnaire to investigate the general empathy, empathy concern (EC), subjective discomfort, and opinion-taking abilities of different therapists, and they found that novices and expert therapists showed significant differences in EC but no significant differences in their capacity for other empathy.

This lack of conformity was further explored in some cortex imaging studies. Recent studies used fMRI to record brain activation in subjects who developed empathy and found that when the individual developed empathy for the pain of others, the cortex activation was highly consistent with when the individual felt his/her own pain (Jackson et al., 2006b; Bernhardt and Singer, 2012; Grice-Jackson et al., 2017; Cogoni et al., 2018). Hence, it was further inferred that individuals who develop empathy from the suffering of others also experience a corresponding pain and discomfort (Lamm et al., 2007). In terms of medical scenarios, to avoid discomfort, medical professionals consciously lower their own empathy (Roter et al., 1997; Suchman et al., 1997). Nevertheless, these studies did not delve into the causes of the decline in empathy of medical professionals, and they often used measures such as scales to determine the empathy of different groups of subjects (Ahrweiler et al., 2014). Given the limitations of the scale method itself, it is impossible to accurately determine the factors that affect the change in empathy of medical professionals (Boynton and Greenhalgh, 2004).

This study uses functional near-infrared spectroscopy (fNIRS) to explore the differences in cortex activation patterns between medical professionals and non-medical professionals when they experienced empathy in medical scenarios. fNIRS has the characteristics of good ecological validity, high temporal resolution and high spatial resolution. This method can record the cortex activation patterns of subjects in natural situations, thus ensuring the high ecological validity of a study (Hoshi et al., 2001; Jang et al., 2009). The experiments required subjects with different professional backgrounds to observe pictures of medical scenarios and to rate pain levels of the pictures, while the cortex activation was monitored by the near-infrared imaging system. Some studies show evidence that medical professional knowledge is responsible for the differences in empathy between medical and non-medical workers (Roter et al., 1997; Suchman et al., 1997; Ahrweiler et al., 2014), and some neuroimaging findings suggest that the experience of one’s own emotion and empathic responses to that of the others may share common neural mechanisms (Avenanti et al., 2006; Gu and Han, 2007; Saarela et al., 2007). We assume that an oxygenated hemoglobin (HbO) curve would be different between medical professionals and non-medical professionals.

## Materials and Methods

### Participants

Sixteen healthy doctoral subjects were recruited from The University of Traditional Chinese Medicine, who had all been trained in acupuncture for more than 3 years and had more than 2 years of clinical experience in acupuncture, aged between 24 and 32 years (mean ± SD: 28.32 ± 1.73), and participated in the study as paid volunteers. Sixteen healthy normal university students (seven females, age mean ± SD: 25.27 ± 3.16) were enrolled as the control group, which ensured that they had no prior acupuncture-related knowledge or experience. All subjects had neither neurological nor psychiatric history. All were right-handed, had normal or corrected-to-normal vision, and were not color blind. Informed consent was obtained from all participants before the experiment. This study was approved by a local ethics committee.

### Stimuli and Questionnaire

#### Acupuncture Images

**FIGURE 1:**
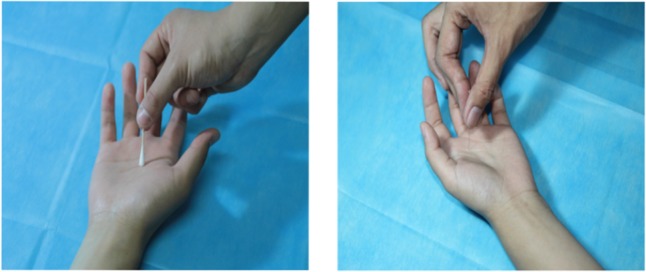
Stimuli examples. Illustration of the stimuli used in the current study. The left picture shows swab contact, and the right picture shows acupuncture.

The visual stimuli consisted of 60 acupuncture scenario pictures that were taken in the clinic, and the background was a light blue sterile draping. Acupuncture was performed in the hand, foot, elbow and knee in three males and three females by graduate students in acupuncture with 3 years of clinical acupuncture experience, and the acupoint was selected according to *Acupuncture and Moxibustion* (ninth edition). Disinfection, acupuncture and needle retention during the acupuncture process were photographed, for a total of 1000 images. After the initial screening, the degree of pain expressed in the images was scored using an 8-point scale by 50 non-medical college students: the higher the score, the more severe the pain they felt for the characters in the images. Images that were taken in the acupuncture setting and scored higher than 6 points (*M* = 6.35, SD = 1.07) and those that were taken in the swab contact scene and scored lower than 2 points (*M* = 1.87, SD = 1.25) were selected as the experimental material. Finally, a total of 30 images were included, as shown in **Figure 1**.

#### Interpersonal Reactivity Index-C Questionnaire

The Interpersonal Reactivity Index-C (IRI-C) questionnaire, which was revised by Zhang et al. (2010) on the basis of IRI (Davis, 1980), was used to measure the empathic ability of participants. The questionnaire consisted of 22 questions divided into four subscales: perspective taking (PT, the tendency to adopt the point of view of other people), EC (the tendency to experience feelings of warmth, compassion and concern for other people), personal distress (PD, one’s own feelings of personal unease and discomfort in reaction to the emotions of others) and fantasy (FS, an exciting and unusual experience or situation you imagine happening, but which will probably never happen). Cronbach’s alpha of IRI-C was 0.750, and Cronbach’s alpha of PT, EC, PD, and FS was 0.721, 0.532, 0.758, and 0.624, respectively.

### Design and Procedure

This study adopted a block design that was a mixed experiment of 2 (material type: swab contact vs acupuncture scenarios) × 2 (subject group: medical professional vs non-medical professional), and it consisted of eight blocks, each block including 15 images. The material type referred to the medical practice in which the character is admitted, including undergoing swab contact and being punctured by acupuncture needles.

**FIGURE 2:**
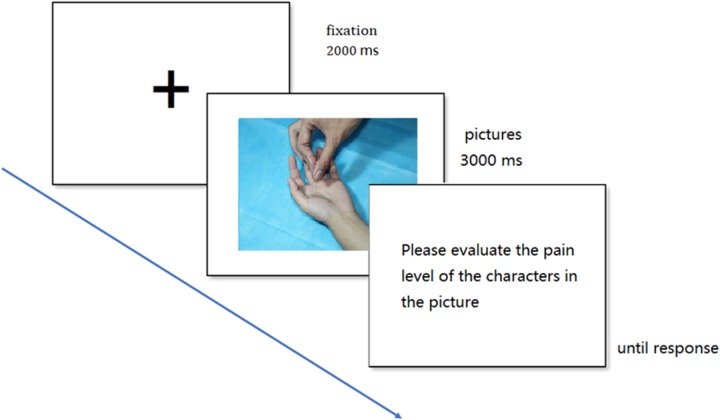
Example of trial events. Each trial displayed the fixation cross (2000 ms) followed by the experimental picture (3000 ms) and the evaluation question, which remained until a response occurred.

The experiment was conducted in a quiet room. Subjects were sitting in front of a CRT monitor, with their eyes right in the center of the screen at a distance of 70 cm. In the experiment, blocks were presented in a random sequence. At the beginning of each trial, the fixation point was presented for 2000 ms, then, the experimental picture was presented for 3000 ms. After the picture disappeared, an evaluation screen was presented and the subjects were asked to rate the pain of the model in the picture using the 8-point scale, where the 1–8 scores, respectively, corresponded to the “A, S, D, F, G, H, J, K” keys on the computer keyboard, followed by the next trial. During the presentation process, a continuous performance task was added to each block, aiming to randomly add a marker on the fixation point page, and the subjects were asked about the number of markers at the end of each block to make the subjects maintain a high degree of attention. The experimental procedure is shown in **Figure 2**. After the experiment, all the subjects were asked to fill in the IRI-C questionnaire.

### fNIRS Data Acquisition

Stimulus presentation and behavioral data acquisition relied on E-prime 2.0 (Windows XP). Stimuli were presented on a 21-inch CRT monitor with a screen refresh rate of 85 Hz. Cerebral oxygenation changes were sampled by a 52-channel fNIRS system (LABNIRS/16, Shimadzu Corporation, Kyoto, Japan). The system is a continuous-wave device that measures changes in attenuation at three wavelengths (780 ± 5, 805 ± 5, and 830 ± 5 nm). Equipped with 16 light-emitting and 16 detector probes, 52 channels can be measured quasi-simultaneously. Concentration changes of HbO and deoxygenated hemoglobin (Hb) were measured simultaneously, and changes of total Hb were calculated by adding HbO and Hb (Huppert et al., 2006; Chang et al., 2014). Optical data were transformed into HbO and Hb according to the modified Beer–Lambert law (Baker et al., 2014). The blood oxygen concentration was calculated by referring to the algorithm used in spm_fnirs (Duncan et al., 1996; Homae et al., 2007; Scholkmann et al., 2014).

The current study is focused on the detection of the sensory cortex in the prefrontal area (Lamm et al., 2007) and left soma (Bolognini et al., 2013). Lamm et al. (2007) reported that the left postcentral gyrus was activated in non-medical professionals when they viewed acupuncture pictures, whereas the sensory cortex was activated in the prefrontal area but not in the soma in medical professionals. Bolognini et al. (2013) found that the sensory cortex in the left soma was associated with the empathy situation that was not related to physical contact; by contrast, the sensory cortex in the right soma was associated with the empathy situation related to physical contact (Bolognini et al., 2013).

**FIGURE 3:**
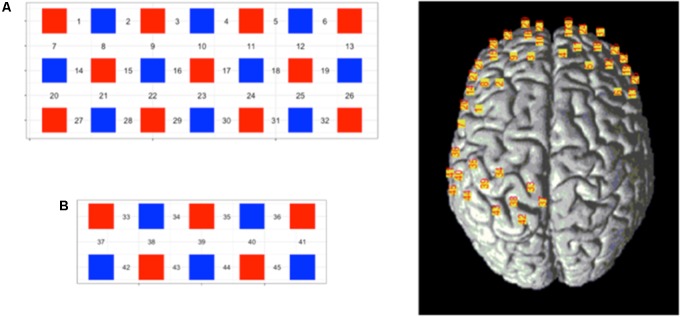
Schematic representation of the 45-channel fNIRS probe sets placed on the participants’ scalps, separated into the frontal lobe [**(A)** block, channels: 1—32] and occipital lobe [**(B)** block, channels: 33–45]. Red squares represent light emitters, blue squares represent detectors, and numbers represent the measurement channels. Cranial optode channels in relation to the underlying anatomical structures are shown for one representative subject.

The channel was designed to have two blocks. Block A was a 7 × 3 layout consisting of 11 detectors and 10 receivers, whereas block B was a 5 × 2 layout consisting of 5 transmitters and 5 receivers, the interoptode distance was 3 cm. The probe layout and channel distribution in the brain are shown in **Figure 3**. According to the 10–20 system, two withdrawals were used to cover the corresponding sites; the position of each probe was located using a 3D locator and calibrated through the standard MNI coordinate to obtain the correspondence between the channel locations and the Broudman partitions. The regions of interest were the sensory cortex in the prefrontal lobe and soma.

### fNIRS Data Analysis

The general linear model (GLM) analysis was performed for each participant. GLM is generally used in fMRI studies (Friston et al., 1994; Scholkmann et al., 2010). The GLM analysis was performed as follows: for each participant, the hemodynamic response function (HRF) filter and a wavelet-minimum description length (MDL) detrending algorithm were used to remove physical noise and artifacts, and a baseline correction was performed. After the wavelet-MDL-based detrending, the average HbO time series were estimated by integrating each HRF with the relevant experimental paradigms (Cai et al., 2017). In the current study, GLM can describe a measurement of change in HbO in terms of a linear combination of two conditions (swab contact and acupuncture) and two groups (medical professional and non-medical professional). The beta values of the GLM for different trials were extracted as weights to account for the brain activity. Topography (based on the beta values) was plotted based on the location of the channels. We prioritized HbO results because HbO signals were the most sensitive index to reflect cerebral blood flow activities and HbO signals were widely reported (Homae et al., 2006). These analyses were completed by NIRS-SPM and SPSS 22.0. The SPM-based software package for fNIRS data analysis was based on the GLM.

## Results

### IRI-C Questionnaire Scores

The total score and scores of four dimensions for the IRI-C questionnaire of the two groups are listed in **Table 1**. Neither the scores for the different dimensions nor the total score differed significantly between the medical professional and non-medical professional groups.

**Table 1:** IRI score of the medical and non-medical groups.

	Medical group		Non-medical group		*t*	*p*
	*M*	SD	*M*	SD		
Opinion perspective	10.87	3.63	10.00	3.42	0.63	0.53
Personal pain	8.74	4.22	11.25	3.81	−1.62	0.13
Imagination	9.94	4.30	10.25	5.28	−0.15	0.87
Empathy and care	11.10	3.37	11.38	1.92	−0.30	0.76
Total	40.52	11.24	43.12	11.63	−0.56	0.58

### Pain Rating Scores

The pain rating scores for the two groups are shown in **Table 2**. The main effect of subject group is not significant, *F*(1,30) = 0.375, *p* > 0.05; the main effect of material type is significant, *F*(1,30) = 281.312, *p* < 0.001, η^2^ = 0.996; the interact effect of subject group and material type is not significant, *F*(1,30) = 4.202, *p* < 0.05, *η^2^* = 0.510. For the viewing of swab contact pictures, there was no significant difference in pain evaluation scores between the medical professional group (*M* = 1.69) and the non-medical professional group (*M* = 1.64) (*p* > 0.05). For the viewing of acupuncture pictures, the pain evaluation score for the medical professional group (*M* = 4.36) was significantly lower than the score for the non-medical professional group (*M* = 5.05) (*p* < 0.001).

**Table 2:** Pain evaluation scores in the medical and non-medical groups.

	Medical group		Non-medical group	
	*M*	SD	*M*	SD
Swab contact	1.69	1.09	1.64	0.87
Acupuncture	4.36	1.61	5.05	0.66

### fNIRS Results

**FIGURE 4:**
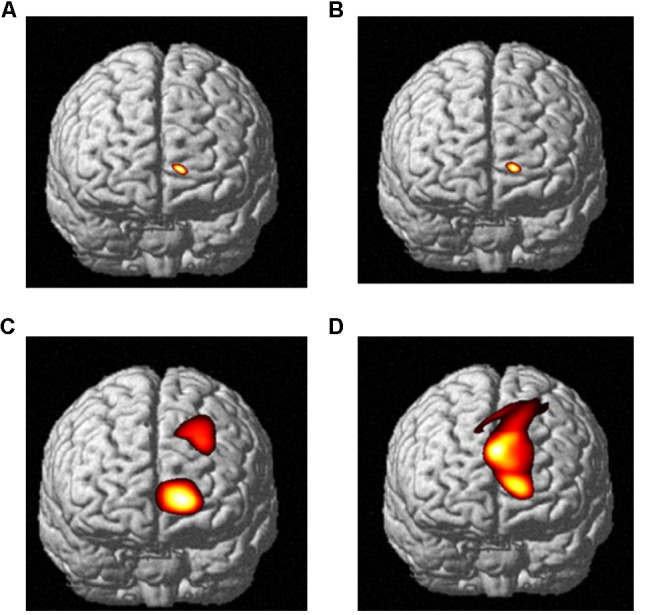
The relative oxyHb concentrations from the DLPFC channels of data obtained from both medical and non-medical subjects under two different conditions. Category: **(A)** medical subjects under the swab contact condition; **(B)** medical subjects under the swab contact condition; **(C)** medical subjects under the acupuncture condition; and **(D)** non-medical subjects under the acupuncture condition.

Near-infrared imaging found that when the subjects observed the pictures of the swab and needle contact with the body, the corresponding regions of the somatosensory cortex in both the medical professionals and the non-medical professionals were significantly correlated with the experimental tasks and somewhat correlated with the frontal lobe (**Table 3** and **Figure 4**). The activated channels of non-medical professionals included 7, 12, 15, 17, 22, 25, 27, 28, 29, 31, 32, 34, 40, 41, 42, and 44, which corresponded to the dorsolateral prefrontal cortex (DLPFC), frontal polar regions, posterior part of the inferior frontal gyrus, supramarginal gyrus, supplementary somatosensory cortex, and angular gyrus, of which nine channels corresponded to the DLPFC. Therefore, this cortical region played an important role in the task. Meanwhile, the activated channels for medical professionals included the somatosensory cortex and the prefrontal cortex.

**Table 3:** Comparison of the β value for each channel between the medical and non-medical groups.

	Medical group		Non-medical group		*t*	*p*
	*M*	SD	*M*	SD		
ch4	1.78e–04	3.09e–04	−3.01e–05	1.10e–04	−2.27	0.037
ch6	2.01e–04	3.70e–04	−3.23e–05	1.25e–04	−2.14	0.049
ch13	1.84e–04	2.70e–04	−8.14e–05	2.03e–04	−2.50	0.023
ch14	1.68e–04	3.07e–04	−2.96e–05	1.10e–04	−2.18	0.045
ch20	3.74e–04	4.60e–04	−1.33e–04	2.34e–04	−3.51	0.002
ch24	3.41e–04	6.68e–04	−9.85e–05	2.79e–04	−2.18	0.044
ch35	4.75e–04	4.81e–04	1.22e–07	4.27e–04	−2.61	0.016
ch37	4.34e–04	7.13e–04	−3.96e–04	1.04e–04	−2.30	0.032

*Only the channel of which there is a significant difference between the medical and non-medical groups is reported.*

**FIGURE 5:**
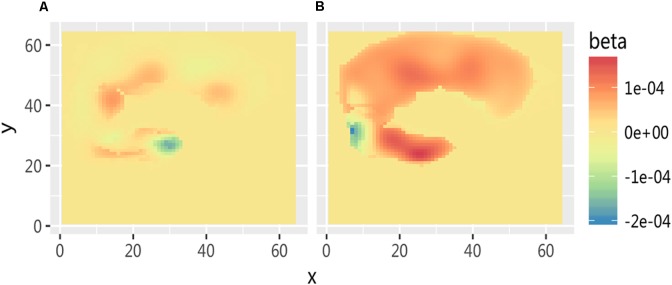
Comparison of cortex activation evoked by stimuli of needle puncture. **(A)** Medical subjects under the acupuncture condition and **(B)** non-medical subjects under the acupuncture condition.

The fNIRS imaging results of cortex activation evoked by the stimuli of needle puncture (**Figure 5**) were submitted to a *t*-test and the results showed that when the two groups observed acupuncture pictures, the activation differences in cerebral function were mainly in the somatosensory cortex and the frontal cortex. It indicates that this area is a cortical area associated with empathy for pain.

## Discussion

This work investigated the modulation of neural correlates of empathy for pain by the professional knowledge of stimulus reality. Similar to the prior study (Jackson et al., 2005, 2006a; Gu and Han, 2007), we identified neural correlates of pain-related empathic responses by contrasting painful and neutral stimuli. The results indicated that there is no significant difference in empathy between the medical professional and non-medical professional groups on questionnaire scores. Questionnaire scores reflect the general characteristics or the more stable abilities of individuals, and these abilities often do not change with the task or situation (Boynton and Greenhalgh, 2004; Graeff, 2005). Meanwhile, the results showed that the two groups differed significantly in their empathy for pain when observing acupuncture pictures. These pain rating results demonstrated that the subjects in both the medical professional and non-medical professional groups did not feel obvious pain when they witnessed the pain of individuals with the swab contact.

Furthermore, under the observation of individuals undergoing acupuncture, the pain rating of medical professional subjects was significantly lower than that of non-medical professional subjects. fNIRS imaging showed that there were three significant brain activation differences between the two groups when they were viewing pictures of medical scenarios. In particular, the first region included the ACC, DLPFC, and other areas in the frontal lobe, which were mainly related to cognitive control function. The second region included the somatosensory cortex and other matrix areas, which were the areas involved in the sensation of pain. The third region was the motor cortex, which mainly executed motor-related functions.

An important finding of this work was that the activity of the left somatosensory cortex in subjects had a high correlation with the experimental tasks regardless of whether the participants observed swab contact or acupuncture pictures. The somatosensory cortex is directly related to the physical sensation of an individual (Purves, 2012). However, studies have shown a significant correlation between the left somatosensory cortex and pain sensation (Bufalari et al., 2007). Benuzzi et al. (2008) revealed that some of the cerebral cortex involved in somatosensory processing, such as part of the parietal cortex and the left posterior cerebral insula cortex, is also activated during empathy for pain. A subsequent study by Bolognini et al. (2013) found that the right somatosensory cortex was associated with empathy in situations related to physical contact, whereas the left somatosensory cortex was associated with empathy in situations related to nonphysical contact. The results of this experiment further confirmed that the somatosensory cortex appeared to be associated with activation when subjects observed the simulation of physical contact. Hence, although the somatosensory cortex is involved in feeling empathy for pain, it is not a specific area for feeling this type of empathy.

The DLPFC is known for its involvement in the executive functions, where it is mainly involved in the control and regulation of emotion and behavior in the neuropsychological mechanism of empathy (Behrens et al., 2007). In this study, this area was significantly more activated in medical professionals compared with non-medical professionals. Thus, it can be inferred that medical professionals adjust and control their own ability to feel empathy for pain. Because the area is primarily responsible for high-level executive function, it is primarily responsible for the high-level, top–down cognitive control and emotional regulation in the empathy process (Goubert et al., 2005; Wu et al., 2015). Therefore, the activation of the DLPFC modulates the subjective feelings of empathy of the subjects for pain, which in turn affects the score for the pain stimulating picture but does not have an essential effect on the empathy ability of an individual (Grice-Jackson et al., 2017). A previous study confirmed that ACC was common for rating painful pictures, which indicated that ACC was engaged in cognitive evaluation of pain of another (Gu and Han, 2007). According to Goubert et al. (2005), empathy depends on both bottom–up and top–down (observers’ knowledge) processes. Thus, medical subjects’ prior knowledge of the medical stimuli reduced empathetic responses to others’ pain. In other words, the top–down process (the prior knowledge of medicine) may contribute to the neural activity that distinguishes empathy for pain of medical scenarios.

The motor cortex is mainly responsible for planning, control, and implementation of autonomous motion (Shima and Tanji, 1998). Studies have shown that high-level athletes activate similar areas when planning and imagining their movements (Moro et al., 2013). In this work, the motor cortex presented a high degree of activation in medical professionals specializing in acupuncture when they observed acupuncture pictures. This result may have been observed because after a long period of professional training, these subjects automatically start to process the simulation of the acupuncture process when they watch the acupuncture scenes, thus causing the activation of the corresponding brain area. In summary, the results demonstrate that medical professionals experience less empathy for pain in medical scenarios, and brain activation patterns indicate that the empathy process is subject to the influence of top–down processing of medical professional knowledge.

## Ethics Statement

This study was carried out in accordance with the recommendations of Ethical guidelines for scientific research, the research ethics committee of the Academy of Psychological and Behavioral Research, Tianjin Normal University. The protocol was approved by the research ethics committee of the Academy of Psychological and Behavioral Research, Tianjin Normal University. All subjects gave written informed consent in accordance with the Declaration of Helsinki.

## Author Contributions

JX and XX designed and conducted the experiment, analyzed the data, and wrote the manuscript. HY and XX contributed to data collection and manuscript revision. JX and SY contributed to manuscript revision.

## Conflict of Interest Statement

The authors declare that the research was conducted in the absence of any commercial or financial relationships that could be construed as a potential conflict of interest.
